# Dysfunction of Corticomotoneurons in Guillain-Barré Syndrome (GBS)?

**Published:** 2009-10-01

**Authors:** Steve Vucic

**Affiliations:** Department of Neurology, Westmead Hospital, Western Clinical School, University of Sydney, Wentworthville, NSW, 2145, Sydney, Australia. Email: s.vucic@powmri.edu.au

**Keywords:** conduction block, Guillain-Barré syndrome, TMS

## Abstract

Guillain-Barré syndrome (GBS) is characterized by acute and symmetric flaccid paraparesis and areflexia. Involvement of the central nervous system has been infrequently reported. In the current issue of Clinical Medicine: Case reports, an unusual case of GBS with asymmetric muscle weakness was reported. Corticomotoneuronal dysfunction was invoked as a possible cause for this neurological finding. Reversible blockade of voltage gated Na^+^ channels resulting in conduction failure may be a possible pathophysiological mechanism.

Guillain-Barré syndrome (GBS) is a rare autoimmune neuroinflammatory disorder of the peripheral nerves clinically characterized by acute and symmetric flaccid paraparesis with areflexia.^1^ Although traditionally regarded as a peripheral nerve disorder, inflammation within the central nervous system (CNS) has been rarely reported in GBS.^2^ Specifically, degeneration of spinal cord dorsal columns as well as mononuclear cell infiltrates consisting of lymphocytes and macrophages were reported in the spinal cord and brainstem of GBS patients. Further, diffuse and focal activation of microglia was also reported in the spinal cord, brainstem and periventricular regions of the CNS. These changes were similar to the inflammatory changes seen in the peripheral nerves and were interpreted as representing either secondary changes or CNS immune activation in response to an unidentified antigen.^2^

Of further relevance, dysfunction of corticospinal tracts, as reflected by prolonged central motor conduction time (CMCT), was reported in a cohort of patients with acute paralysis, hyper-reflexia and the presence of anti-ganglioside antibodies including GM1/GM2, GT1a, GT1b and GD1b.^3^ This prolongation in central motor conduction time rapidly improved with intravenous immunoglobulin (IVIg) treatment and paralleled the clinical improvement, thereby suggesting that an antibody-mediated process was the underlying pathophysiology. However, in GBS patients with the acute motor axonal neuropathy sub-type, central motor conduction time was normal, thereby arguing against dysfunction within the corticospinal tracts.

In the clinical setting, CNS function may be assessed by using non-invasive transcranial magnetic stimulation (TMS) techniques.^4^ Specifically, central motor conduction time reflects the time of conduction along the corticospinal tract between the motor cortex and spinal motor neuron.^4^,^5^ Factors contributing to generation of the central motor conduction time include the time to activate the cortical pyramidal cells, conduction time of the descending volley along the corticospinal tract, synaptic transmission and activation of spinal motor neurons, peripheral motor axon conduction and neuromuscular transmission time.^6^ In addition to CMCT, CNS function may be assessed by measuring the motor evoked potential (MEP) amplitude which reflects the density of corticomotoneuronal projections onto the spinal motor neuron.^7^ In central demyelinating diseases, such as multiple sclerosis, CMCT is classically prolonged while the MEP amplitude may be attenuated.^4^

In an attempt to further clarify whether CNS dysfunction could contribute to the development of symptoms in GBS, Kiriyama and colleagues in the current issue of Clinical Medicine: Case reports,^8^ describe an unusual case of GBS with asymmetric weakness and reduced deep tendon reflexes in which CNS dysfunction is invoked as a possible cause for the neurological findings. Specifically, CNS dysfunction was suggested by the finding of absent MEP responses in the setting of normal peripheral nerve conduction studies, including cervical nerve root stimulation and F-wave persistence and latencies. Further, the MEP amplitude improved with IVIg treatment, resulting in prolongation of the CMCT, which normalized after one year. The pathophysiological mechanisms underlying this CNS dysfunction may include reversible dysfunction of voltage gated Na^+^ channels at the nodes of Ranvier, possibly mediated by anti-GM1 antibodies, thereby resulting in conduction failure.^9^–^12^ In addition, remyelination of the corticopsoinal axons may have contributed to transient CMCT prolongation. The absence of lesions on brain MRI may not discount CNS demyelination since TMS studies may be more sensitive at detecting smaller demyelinating lesions.^4^ The frequency of CNS involvement in GBS and the mechanisms by which the aberrant autoimmune system mediates CNS dysfunction in GBS patients remains to be determined.
